# Pediatric Patients Hospitalized With Eating Disorders in Ontario, Canada, Over Time

**DOI:** 10.1001/jamanetworkopen.2023.46012

**Published:** 2023-12-04

**Authors:** Sarah Smith, Alice Charach, Teresa To, Alene Toulany, Kinwah Fung, Natasha Saunders

**Affiliations:** 1 Dalla Lana School of Public Health, University of Toronto, Toronto, Canada; 2 ICES, Toronto, Canada; 3 Department of Psychiatry, The Hospital for Sick Children, Toronto, Canada; 4 Department of Psychiatry, Temerty Faculty of Medicine, University of Toronto, Toronto, Canada; 5 Child Health Evaluative Sciences, The Hospital for Sick Children Research Institute, Toronto, Canada; 6 Department of Pediatrics, Temerty Faculty of Medicine, University of Toronto, Toronto, Canada; 7 Edwin S. H. Leong Centre for Healthy Children, University of Toronto, Toronto, Canada

## Abstract

**Importance:**

Understanding the evolving characteristics of pediatric patients hospitalized for eating disorders is important to ensure that services and treatments align with patient needs.

**Objective:**

To examine temporal trends in the rates of hospitalizations for pediatric eating disorders by clinical and demographic characteristics in Ontario, Canada, over a 17-year period.

**Design, Setting, and Participants:**

This population-based, repeated, cross-sectional study used linked health administrative and demographic databases in Ontario, Canada, to identify individuals aged 5 to 17 years hospitalized with eating disorder diagnoses from April 1, 2002, to March 31, 2020. Data analyses were performed from May 2021 to June 2023.

**Exposure:**

Fiscal year (April 1-March 31) of eating disorder hospitalization.

**Main Outcomes and Measures:**

Outcomes of interest were absolute and relative changes in pediatric eating disorder hospitalization rates overall and stratified by patient sex, age groups, and eating disorder diagnostic groups.

**Results:**

Over the study period, there were 11 654 pediatric eating disorder hospitalizations, of which 5268 (45.2%) were for anorexia nervosa and 1374 (11.8%) were for bulimia nervosa. There were a total of 10 648 hospitalizations (91.4%) among female patients, and the median (IQR) age was 15.0 (14-0-16.0) years. Hospitalization rates increased 139% from 2002 to 2019, from 2.0 per 10 000 population to 4.8 per 10 000 population. The largest relative changes were observed among male patients (416%; from 0.2 per 10 000 population to 1.1 per 10 000 population), individuals aged 12 to 14 years (196%; from 2.2 per 10 000 population to 6.6 per 10 000 population), and individuals with eating disorders other than anorexia or bulimia nervosa (255%; from 0.6 per 10 000 population to 2.1 per 10 000 population). Male patients, younger adolescents, and individuals with other eating disorders also represented larger proportions of hospitalizations by fiscal 2019.

**Conclusions and Relevance:**

In this cross-sectional study of eating disorder hospitalizations, pediatric hospitalizations increased over time, particularly among populations traditionally considered atypical. Existing eating disorder programs must adapt to accommodate changing patient presentations and increased volumes to ensure effective care delivery.

## Key Points

**Question:**

How did the characteristics of pediatric patients hospitalized with eating disorder diagnoses in Ontario, Canada, change from 2002 to 2019?

**Findings:**

In this cross-sectional study including 11 654 pediatric eating disorder hospitalizations, rates of pediatric eating disorder hospitalizations increased 139% from 2002 to 2019. The largest relative changes in rates were for males, individuals aged 12 to 14 years, and individuals with eating disorders other than anorexia or bulimia nervosa.

**Meaning:**

These findings underscore the necessity of tailored treatment approaches and programs to address the increasing care needs within these different subpopulations of pediatric patients with eating disorders.

## Teaser

This cross-sectional study examines temporal trends in the rates of pediatric eating disorder hospitalizations by clinical and demographic characteristics in Ontario, Canada, over a 17-year period.

## Introduction

Eating disorders are serious psychiatric illnesses characterized by patterns of restrictive or excessive eating, often accompanied by harmful behaviors, such as self-induced vomiting or excessive exercise. Typically originating in childhood or adolescence,^1^ these disorders are estimated to affect between 6% and 13% of adolescents.^2,3^ Eating disorders are associated with high rates of medical complications,^4^ psychiatric and medical comorbidity,^5^ functional impairment,^2^ family distress,^6^ and some of the highest mortality rates of all psychiatric illnesses.^7^ Canadian practice guidelines recommend hospitalization to stabilize pediatric patients whose condition is too severe to be managed safely in alternative treatment settings.^8^

Prior research has demonstrated a concerning upward trend in rates of pediatric hospitalizations with eating disorder diagnoses in Ontario, Canada.^9^ Similar observations have been made in Europe and the United States.^10,11,12^ However, little is known about the health service use of pediatric patients with eating disorders who have traditionally been considered atypical, including males, children younger than 12 years, and patients with eating disorders other than anorexia or bulimia nervosa, such as avoidant restrictive food intake disorder and other specified feeding and eating disorders.^13,14,15,16,17,18,19,20,21,22,23^ International research using health administrative databases has shown increases in eating disorder treatment for pediatric patients with some of these characteristics over time.^11,12,24^ To our knowledge, the health service use of these patient groups has not been formally evaluated in Canada but is important for service planning, health care practitioner education, and clinical research, given the scarcity of evidence on how to best treat these young patients.

The primary objective of this study was to examine temporal trends in rates of hospitalizations for children and adolescents with eating disorder diagnoses by sex, age group, and eating disorder diagnoses. The proportions of hospitalizations associated with these characteristics in 2002 and 2019 are also compared. We hypothesized that relative increases in rates of eating disorder hospitalizations would be largest for males, young children (age <12 years), and eating disorder diagnoses other than anorexia nervosa or bulimia nervosa.

## Methods

This cross-sectional study used data authorized under section 45 of Ontario’s Personal Health Information Protection Act and is exempt from review by a research ethics board.^25^ Provincial legislation allows ICES to collect and analyze health care data without individual patient consent for health system evaluation. This study followed the Strengthening the Reporting of Observational Studies in Epidemiology (STROBE) reporting guideline and the Reporting of Studies Conducted Using Observational Routinely-Collected Health Data (RECORD) Statement.

### Study Design, Setting, and Population

This was a population-based, repeated cross-sectional study using linked health administrative databases housed at ICES, an independent not-for-profit research institute in Ontario, Canada. Ontario is Canada’s largest province, representing approximately 40% of Canada’s population. All children and adolescents eligible for provincial health insurance between April 1, 2002 (fiscal year [FY] 2002), and March 31, 2020 (FY 2019), were included. In Canada, an FY is from April 1 to March 31 of the following year. Data were linked using unique encoded identifiers and analyzed at ICES.

### Data Sources

We identified provincial residents’ age, sex, and postal code using the provincial health insurance registry (Ontario Registered Persons Database). We extracted hospitalizations with eating disorder diagnoses and other diagnoses contributing to the hospital stay using the Canadian Institute for Health Information’s Discharge Abstract Database (CIHI-DAD) and the Ontario Mental Health Reporting System (OMHRS). Canadian Census data from 2001, 2006, 2011, and 2016 (using the Statistics Canada postal code conversion files at ICES) were used to determine whether individuals resided in rural communities.^26^

### Outcome Measure and Exposure

The primary outcome was the annual rate of hospitalization of individuals aged 5 to 17 years with eating disorder diagnoses per 10 000 population in Ontario from FY 2002 to FY 2019. Overall rates and specific rates by sex (male and female), age group (<12, 12-14, and 15-17 years), and eating disorder diagnoses (anorexia nervosa, bulimia nervosa, or other eating disorder) were calculated. Other eating disorders included eating disorders not otherwise specified, pica, rumination, and feeding disorders of childhood. Hospitalizations with eating disorder diagnoses were defined using diagnostic codes from the *International Statistical Classification of Diseases and Related Health Problems, Tenth Revision* (*ICD-10*) with Canadian modifications, the *Diagnostic and Statistical Manual of Mental Disorders* (Fourth Edition) (*DSM-IV*)^27^ and the *Diagnostic and Statistical Manual of Mental Disorders* (Fifth Edition) (*DSM-5*)^28^ in CIHI-DAD (codes F500, F501, F502, F503, F504, F505, F508, F509, F982, and F983) or OMHRS (codes 307.1, 307.50, 307.51, 307.52, 307.53, 307.59, and provisional diagnoses 10 and 12). The feasibility of identifying eating disorder hospitalizations using *ICD-10* and *DSM-IV* and *DSM-5* codes in CIHI-DAD and OMHRS databases has been established in prior Canadian research.^29^ Specific codes used are available in eTable 1 in Supplement 1. For eating disorder diagnostic groupings, hospitalizations were sorted into mutually exclusive groups. The *DSM-IV* and *DSM-5* detail mutually exclusive diagnostic criteria for many diagnoses, such that individuals cannot have multiple eating disorder diagnoses concurrently. Specifically, a *DSM-IV* or *DSM-5* diagnosis of anorexia nervosa supersedes a diagnosis of bulimia nervosa and a *DSM-IV* or *DSM-5* diagnosis of anorexia or bulimia nervosa supersedes the other eating disorder diagnoses included in this study. Hospitalizations of individuals younger than 5 years, older than 17 years, and not residing in Ontario were excluded. Annual population counts of individuals aged 5 to 17 years eligible for provincial insurance from Ontario’s health system registry were used as the population denominators.^30^

### Covariates

For each hospitalization, we measured the number of co-occurring psychiatric disorders identified during that hospitalization and defined using *ICD-10*, *DSM-IV*, and *DSM-5* diagnostic codes (eTable 2 in Supplement 1) to support understanding of potential changes in complexity (ie, greater number of co-occurring psychiatric disorders) of patients over time. We identified individual-level patient rural and urban residence, in which we defined urban as residing in a census metropolitan area (CMA) or census agglomeration (CA) with at least 10 000 residents.^26^ We measured socioeconomic status using Statistics Canada’s national neighborhood income quintiles, ranging from lowest (first) to highest (fifth).^31^ Urban-rural residence and socioeconomic status were included to provide context about sociodemographic factors that may have contributed to findings. We then measured length of stay, defined as the number of days between admission and discharge (inclusive), to provide context for health system planning.

### Statistical Analysis

The demographic and clinical characteristics of hospitalized children and adolescents are presented using descriptive statistics with hospitalization as the unit of analysis; therefore, the same individual patient may have contributed to multiple hospitalizations. Annual and relative percentage changes in rates of pediatric eating disorder hospitalizations were calculated per 10 000 specified population overall and by patient sex, age, and diagnostic group. The proportion of hospitalizations with these characteristics in 2002 and 2019 were compared using standardized differences^32^ with results greater than 0.1 (10%) considered meaningful.

Analyses were conducted using SAS software version 9.4 (SAS Institute). Data were analyzed from May 2021 to June 2023.

## Results

### Baseline Characteristics

Of 11 654 pediatric hospitalizations for eating disorders included during the study period (eFigure 1 in Supplement 1), most (10 648 hospitalizations [91.4%]) were for female patients. There were 7550 hospitalizations (64.8%) for adolescents aged 15 to 17 years and 628 hospitalizations(5.4%) for children younger than 12 years; the median (IQR) age was 15.0 (14.0-16.0) years. Most hospitalizations were for anorexia nervosa (5268 hospitalizations [45.2%]) followed by other eating disorders (5012 hospitalizations [43.0%]) and bulimia nervosa (1374 hospitalizations [11.8%]). Most hospitalizations were for children living in urban areas (10 469 hospitalizations [89.8%]) and in the highest neighborhood income quintile (3478 hospitalizations [29.8%]) (Table 1). There were no missing data for patient sex, age at discharge, or eating disorder diagnostic codes. Less than 1% of hospitalizations had missing data on rurality or neighborhood income quintile (Table 1).

**Table 1.** Demographic and Clinical Characteristics of Children and Adolescents Hospitalized for Pediatric Eating Disorders From 2002 to 2019 in Ontario, Canada

Characteristic	Hospitalizations, No. (%) (N = 11 654)
Age, y	
Median (IQR)	15.0 (14.0-16.0)
<12	628 (5.4)
12-14	3476 (29.8)
15-17	7550 (64.8)
Sex	
Male	1006 (8.6)
Female	10 648 (91.4)
Eating disorder diagnostic category	
Anorexia nervosa	5268 (45.2)
Bulimia nervosa	1374 (11.8)
Other eating disorder^a^	5012 (43.0)
Co-occurring psychiatric diagnoses contributing to hospital stay, median (IQR), No.	1.0 (0.0-3.0)
Rurality	
Rural	1155 (9.9)
Urban	10 469 (89.8)
Missing	30 (0.3)
Neighborhood income quintile	
1 (Low)	1676 (14.4)
2	1971 (16.9)
3	2082 (17.9)
4	2393 (20.5)
5 (High)	3478 (29.8)
Missing	54 (0.5)
Length of stay, median (IQR), d	12 (5-29)

^a^ Other eating disorder includes eating disorder not otherwise specified, other eating disorder, unspecified eating disorders, pica, rumination, psychological vomiting, psychological overeating, feeding disorders of childhood, and provisional eating disorders.

### Trends in Overall Hospitalizations Rates

Over 17 years, the annual rate of eating disorder hospitalizations increased 139% (2.8 hospitalizations per 10 000 population), from 2.0 hospitalizations per 10 000 population (424 total hospitalizations) in 2002 to 4.8 hospitalizations per 10 000 population (963 total hospitalizations) in 2019. Large absolute and relative increases were observed, particularly from 2010 to 2013, with the peak in 2013 at 5.1 hospitalizations per 10 000 population (Table 2, Table 3, Table 4, and the Figure). During this period, there was no discernable change in length of stay, although the number of co-occurring psychiatric diagnoses per hospitalization increased (standardized difference, 0.53).

**Table 2.** Comparison of Demographic and Clinical Characteristics of Children and Adolescents Hospitalized for Pediatric Eating Disorders in 2002 and 2019 in Ontario, Canada

Characteristic	2002 Hospitalizations (n = 424)		2019 Hospitalizations (n = 963)		Standardized difference^a^
	No. (%)	No. per 10 000 population	No. (%)	No. per 10 000 population	
Age, y					
<12	26 (6.1)	0.2	59 (6.1)	0.6	0.00
12-14	110 (25.9)	2.2	307 (31.9)	6.6	0.13
15-17	288 (67.9)	5.9	597 (62.0)	12.6	0.12
Sex					
Male	23 (5.4)	0.2	114 (11.8)	1.1	0.23
Female	401 (94.6)	3.9	849 (88.2)	8.7	0.23
Eating disorder diagnostic category					
Anorexia nervosa	216 (50.9)	1.0	429 (44.5)	2.2	0.09
Bulimia nervosa	83 (19.6)	0.4	83 (8.6)	0.4	0.32
Other eating disorder^b^	125 (29.4)	0.6	451 (46.8)	2.1	0.36
Co-occurring psychiatric diagnoses contributing to hospital stay, mean (SD), No.	0.8 (1.1)	NC	1.6 (1.6)	NC	0.53
Rurality					
Rural	49 (11.6)	NC	94 (9.8)	NC	0.06
Urban	375 (88.4)	NC	867 (90.0)	NC	0.05
Neighborhood income quintile					
1 (Low)	54 (12.7)	NC	153 (15.9)	NC	0.09
2	79 (18.6)	NC	144 (15.0)	NC	0.10
3	63 (14.9)	NC	206 (21.4)	NC	0.17
4	90 (21.2)	NC	212 (22.0)	NC	0.02
5 (High)	138 (32.5)	NC	240 (24.9)	NC	0.17
Length of stay, median (IQR), d	11 (4-36)	NC	11 (5-20)	NC	0.06

Abbreviation: NC, not calculated.

^a^ Standardized differences compare covariates independent of sample size. Values larger than 0.1 represent imbalance.

^b^ Other eating disorder includes eating disorder not otherwise specified, other eating disorder, unspecified eating disorders, pica, rumination, psychological vomiting, psychological overeating, feeding disorders of childhood, and provisional eating disorders.

**Table 3.** Annual Rates of Pediatric Hospitalization With Eating Disorder Diagnoses per 10 000 Population and Percent Change in Rates by Patient Characteristics From 2002 to 2019

Characteristics	Hospitalizations (N = 11654)																	
	2002	2003	2004	2005	2006	2007	2008	2009	2010	2011	2012	2013	2014	2015	2016	2017	2018	2019
**Overall**																		
Hospitalizations, No.	424	455	392	405	406	414	431	405	440	608	808	1018	934	872	866	862	951	963
Rate, No. per 10 000 population	2.0	2.2	1.9	1.9	1.9	2.0	2.1	2.0	2.2	3.0	4.1	5.1	4.8	4.5	4.4	4.4	4.8	4.8
Annual change, %	NA	7.5	−13.9	3.8	0.5	3.1	5.0	−5.7	9.6	39.2	34.1	26.9	−7.6	−6.3	−0.9	−1.1	9.6	0.6
Relative change, %	0^a^	7.5	−7.5	−4.0	−3.5	−0.5	4.5	−1.5	7.7	50.3	101.5	155.7	136.3	121.4	119.4	116.9	137.8	139.3
**Male patients**																		
Hospitalizations, No.	23	35	23	34	40	42	41	44	43	47	56	55	61	67	77	89	116	114
Rate, No. per 10 000 population	0.2	0.3	0.2	0.3	0.4	0.4	0.4	0.4	0.4	0.5	0.5	0.5	0.6	0.7	0.8	0.9	1.1	1.1
Annual change, %	NA	52.1	−34.1	48.1	18.4	5.8	−1.6	7.9	−1.8	9.9	16.0	0.9	13.8	10.3	20.6	9.3	28.4	−2.5
Relative change, %	0^a^	52.1	0.2	48.4	75.7	85.8	82.9	97.4	93.8	113.0	147.0	149.2	183.6	212.7	277.1	312.3	429.5	416.4
**Female patients**																		
Hospitalizations, No.	401	420	369	371	366	372	390	361	397	561	752	963	873	805	789	773	835	849
Rate, No. per 10 000 population	3.9	4.1	3.6	3.6	3.6	3.7	3.9	3.6	4.0	5.7	7.8	10.0	9.1	8.4	8.2	8.0	8.6	8.7
Annual change, %	NA	4.6	−12.1	0.8	−0.7	2.4	5.9	−6.6	10.9	42.4	35.6	28.8	−8.8	−7.5	−2.8	−2.0	7.4	0.9
Relative change, %	0^a^	4.6	−8.1	−7.3	−8.0	−5.8	−0.3	−6.9	3.3	47.0	99.3	156.7	134.0	116.4	110.5	106.2	121.4	123.3
**<12 y**																		
Hospitalizations, No.	26	15	17	16	18	24	31	27	33	38	41	27	44	49	51	50	62	59
Rate, No. per 10 000 population	0.2	0.1	0.2	0.2	0.2	0.2	0.3	0.2	0.3	0.4	0.4	0.3	0.4	0.5	0.5	0.5	0.6	0.6
Annual change, %	NA	−41.6	15.2	−4.4	14.8	35.5	31.0	−12.4	22.7	15.2	7.9	−34.6	62.0	10.7	3.3	−2.78	23.1	−5.5
Relative change, %	0^a^	−41.6	−32.7	−35.7	−26.2	−0.0	30.9	14.8	40.8	62.2	74.9	14.4	85.3	105.1	111.9	106.0	153.7	139.9
**Age 12-14 y**																		
Hospitalizations, No.	110	133	124	136	118	126	125	95	129	164	233	293	253	249	280	288	313	307
Rate, No. per 10 000 population	2.2	2.6	2.4	3.4	2.3	2.5	2.5	1.91	2.7	3.5	5.0	6.4	5.6	5.6	6.3	6.4	6.9	6.6
Annual change, %	NA	17.3	−8.1	39.7	−30.8	6.9	−0.6	−23.1	39.2	30.6	44.7	28.2	−12.5	−0.3	11.7	2.0	7.2	−3.2
Relative change, %	0^a^	17.3	7.9	50.6	4.1	11.3	10.7	−14.8	18.5	54.8	124.0	187.1	151.1	150.4	179.7	185.2	205.7	195.9
**Age 15-17 y**																		
Hospitalizations, No.	288	307	251	253	270	264	275	283	278	406	543	698	637	574	535	524	576	597
Rate, No. per 10 000 population	5.9	6.2	5.0	4.9	5.1	5.0	5.2	5.4	5.3	7.7	10.3	13.9	13.0	11.9	11.2	11.1	12.2	12.6
Annual change, %	NA	6.4	−19.4	−2.1	4.0	−2.6	4.8	3.3	−2.4	46.0	34.1	34.6	−6.1	−8.6	−5.7	−1.52	10.5	2.7
Relative change, %	0^a^	6.4	−14.2	−16.0	−2.2	−14.9	−10.8	−7.9	−10.0	31.3	76.1	136.9	122.5	103.5	91.9	88.9	108.7	114.4
**Anorexia nervosa**																		
Hospitalizations, No.	216	240	203	247	213	219	197	173	169	228	273	422	378	387	426	406	442	429
Rate, No. per 10 000 population	1.0	1.1	1.0	1.2	1.0	1.0	0.9	0.8	0.8	1.1	1.4	2.1	1.9	1.9	2.1	2.0	2.1	2.2
Annual change, %	NA	11.0	−15.3	21.5	−14.1	1.2	−9.6	−11.0	0.0	37.6	19.6	54.8	−9.5	−0.7	11.4	−5.6	6.5	6.6
Relative change, %	0^a^	11.0	−6.0	14.2	1.9	−0.7	−10.2	−20.1	20.1	10.0	31.5	103.5	84.1	82.8	103.7	92.3	104.7	118.3
**Bulimia nervosa**																		
Hospitalizations, No.	83	80	56	45	61	44	63	71	69	91	86	112	120	96	69	85	88	85
Rate, No. per 10 000 population	0.4	0.4	0.3	0.2	0.3	0.2	0.3	0.3	0.3	0.5	0.4	0.5	0.6	0.5	0.3	0.4	0.4	0.4
Annual change, %	NA	−5.0	−29.0	−22.2	38.1	−27.6	47.6	9.7	−5.9	37.5	−2.3	23.3	13.2	−21.7	−29.8	27.3	2.4	−4.7
Relative change, %	0^a^	−5.0	−32.5	−47.5	−27.5	−47.5	−22.5	−15.0	−20.0	10.0	7.5	32.5	50.0	17.5	−17.5	5.0	7.5	2.5
**Other eating disorder^b^**																		
Hospitalizations, No.	125	135	133	113	132	151	171	161	206	290	450	490	439	392	376	374	423	450
Rate, No. per 10 000 population	0.6	0.6	0.6	0.5	0.6	0.7	0.8	0.8	1.0	1.4	2.3	2.5	2.2	2.0	1.9	1.9	2.1	2.1
Annual change, %	NA	6.7	−1.6	−14.3	16.7	15.9	13.7	−4.8	29.1	40.2	57.3	9.8	−9.7	−10.3	−6.0	0.5	12.3	1.4
Relative change, %	0^a^	6.7	5.0	−10.0	5.0	21.7	38.3	31.7	70.0	138.3	275.0	311.7	271.7	233.3	213.3	211.7	250.0	255.0

Abbreviation: NA, not applicable.

^a^ For relative change, 2002 was used as the reference year.

^b^ Other eating disorder indicates eating disorder not otherwise specified, other eating disorder, unspecified eating disorders, pica, rumination, psychological vomiting, psychological overeating, feeding disorders of childhood, and provisional eating disorders.

**Table 4.** Demographic and Clinical Characteristics of Children and Adolescents Hospitalized for Pediatric Eating Disorders From 2002 to 2019

Characteristics	Hospitalizations, No. (%) (N = 11 654)																	
	2002 (n = 424)	2003 (n = 455)	2004 (n = 392)	2005 (n = 405)	2006 (n = 406)	2007 (n = 414)	2008 (n = 431)	2009 (n = 405)	2010 (n = 440)	2011 (n = 608)	2012 (n = 808)	2013 (n = 1018)	2014 (n = 934)	2015 (n = 872)	2016 (n = 866)	2017 (n = 862)	2018 (n = 951)	2019 (n = 963)
Age, y																		
Median (IQR)	15.0 (14.0-17.0)	15.0 (14.0-17.0)	15.0 (14.0-16.0)	15.0 (14.0-16.0)	15.0 (14.0-16.0)	15.0 (14.0-16.0)	16.0 (14.0-16.0)	16.0 (14.0-17.0)	15.0 (14.0-16.0)	15.0 (14.0-16.0)	15.0 (14.0-16.0)	15.0 (14.0-16.0)	15.0 (14.0-16.0)	15.0 (14.0-16.0)	15.0 (14.0-16.0)	15.0 (14.0-16.0)	15.0 (14.0-16.0)	15.0 (14.0-16.0)
<12	26 (6.1)	15 (3.3)	17 (4.3)	16 (4.0)	18 (4.4)	24 (5.8)	31 (7.2)	27 (6.7)	33 (7.5)	38 (6.3)	41 (5.1)	27 (2.7)	44 (4.7)	49 (5.6)	51 (5.9)	50 (5.8)	62 (6.5)	59 (6.1)
12-14	110 (25.9)	133 (29.2)	124 (31.6)	136 (33.6)	118 (29.1)	126 (30.4)	125 (29.0)	95 (23.5)	129 (29.3)	164 (27.0)	233 (28.8)	293 (28.8)	253 (27.1)	249 (28.6)	280 (32.3)	288 (33.4)	313 (32.9)	307 (31.9)
15-17	288 (67.9)	307 (67.5)	251 (64.0)	253 (62.5)	270 (66.5)	264 (63.8)	275 (63.8)	283 (69.9)	278 (63.2)	406 (66.8)	534 (66.1)	698 (68.6)	637 (68.2)	574 (65.8)	535 (61.8)	524 (60.8)	576 (60.6)	597 (62.0)
Sex																		
Male	23 (5.4)	35 (7.7)	23 (5.9)	34 (8.4)	40 (9.9)	42 (10.1)	41 (9.5)	44 (10.9)	43 (9.8)	47 (7.7)	54 (6.7)	54 (5.4)	61 (6.5)	67 (7.7)	81 (9.4)	89 (10.3)	115 (12.2)	113 (11.8)
Female	402 (94.6)	420 (92.3)	369 (94.1)	371 (91.6)	366 (90.1)	372 (89.9)	390 (90.5)	361 (89.1)	397 (90.2)	561 (92.3)	754 (93.3)	964 (94.6)	873 (93.5)	805 (92.3)	785 (90.6)	773 (89.7)	836 (87.8)	850 (88.2)
Diagnostic category																		
Anorexia nervosa	216 (50.9)	240 (52.7)	203 (51.8)	247 (61.0)	213 (52.5)	219 (52.9)	197 (45.7)	173 (42.7)	169 (38.4)	228 (37.5)	273 (33.8)	422 (41.5)	378 (40.5)	387 (44.4)	426 (49.2)	406 (47.1)	442 (46.5)	429 (44.5)
Bulimia nervosa	83 (19.6)	80 (17.6)	56 (14.3)	45 (11.1)	61 (15.0)	44 (10.6)	63 (14.6)	71 (17.5)	65 (14.8)	90 (14.8)	85 (10.5)	106 (10.4)	117 (12.5)	93 (10.7)	64 (7.4)	82 (9.5)	86 (9.0)	83 (8.6)
Other eating disorder^a^	125 (29.5)	135 (29.7)	133 (33.9)	113 (27.9)	132 (32.5)	151 (36.5)	171 (39.7)	161 (39.8)	206 (46.8)	290 (47.7)	450 (55.7)	490 (48.1)	439 (47.0)	392 (45.0)	376 (43.4)	374 (43.4)	423 (44.5)	451 (46.8)
Co-occurring psychiatric diagnoses contributing to hospital stay, median (IQR), No.	0 (0-1)	1 (0-2)	1 (0- 1)	0 (0-1)	1 (0-1)	1 (0-1)	1 (0-1)	1 (0-2)	1 (0-2)	1 (0-2)	1 (0-2)	1 (0-2)	1.0 (0-2)	1 (−2)	1 (0-2)	1.0 (0-2)	1 (0-2)	1 (0-2)
Rurality^b^																		
Rural	49 (11.6)	64 (14.1)	43 (11.0)	46 (11.4)	43 (10.6)	38 (9.2)	41 (9.5)	32 (7.9)	62 (14.1)	61 (10.0)	79 (9.8)	101 (9.9)	86 (9.2)	93 (10.7)	85 (9.8)	63 (7.3)	91 (9.6)	94 (9.8)
Urban	375 (88.4)	391 (85.9)	349 (89.0)	359 (88.6)	363 (89.4)	376 (90.8)	390 (90.5)	373 (92.1)	377 (85.7)	543 (89.3)	728 (90.1)	916 (90.0)	840 (89.9)	775 (88.9)	776 (89.6)	797 (92.5)	857 (90.1)	867 (90.0)
Neighborhood income quintile^b^																		
1 (Lowest)	54 (12.7)	51 (11.2)	60 (15.3)	52 (12.8)	43 (10.6)	39 (9.4)	48 (11.1)	63 (15.6)	61 (13.9)	65 (10.7)	99 (12.3)	137 (13.5)	166 (17.8)	132 (15.1)	151 (17.4)	144 (16.7)	158 (16.6)	154 (15.9)
2	79 (18.6)	77 (16.9)	57 (14.5)	56 (13.8)	79 (19.5)	66 (15.9)	65 (15.1)	68 (16.8)	60 (13.6)	105 (17.3)	139 (17.2)	204 (20.0)	172 (18.4)	166 (19.0)	139 (16.1)	146 (16.9)	149 (15.7)	144 (15.0)
3	63 (14.9)	84 (18.5)	69 (17.6)	57 (14.1)	38 (9.4)	66 (15.9)	79 (18.3)	68 (16.8)	100 (22.7)	122 (20.1)	155 (19.2)	186 (18.3)	124 (13.3)	147 (16.9)	162 (18.7)	165 (19.1)	191 (20.1)	206 (21.4)
4	90 (21.2)	103 (22.6)	83 (21.2)	89 (22.0)	97 (23.9)	86 (20.8)	92 (21.3)	85 (21.0)	95 (21.6)	121 (19.9)	178 (22.0)	195 (19.2)	181 (10.4)	177 (20.3)	166 (19.2)	154 (17.9)	189 (19.9)	212 (22.0)
5 (Highest)	138 (32.5)	140 (30.8)	119 (30.4)	148 (36.5)	146 (36.0)	156 (37.7)	147 (34.1)	121 (29.9)	123 (28.0)	191 (31.4)	235 (29.1)	293 (28.8)	283 (30.3)	245 (28.1)	241 (27.8)	251 (29.1)	261 (27.4)	240 (24.9)
Length of stay, median (IQR), d	11 (4-36)	14 (5-34)	17 (5-41)	18 (7-42)	16 (6-41)	20 (6-48)	14 (4-42)	15 (5-45)	15 (5-42)	11 (4-34)	14 (5-30)	12 (5-27)	11 (5-2)	13 (5-26)	10 (4-21)	11 (5-22)	10 (4-21)	11 (5-20)

^a^ Other eating disorder indicates eating disorder not otherwise specified, other eating disorder, unspecified eating disorders, pica, rumination, psychological vomiting, psychological overeating, feeding disorders of childhood, and provisional eating disorders.

^b^ Missing data added to largest subgroup.

**Figure.**
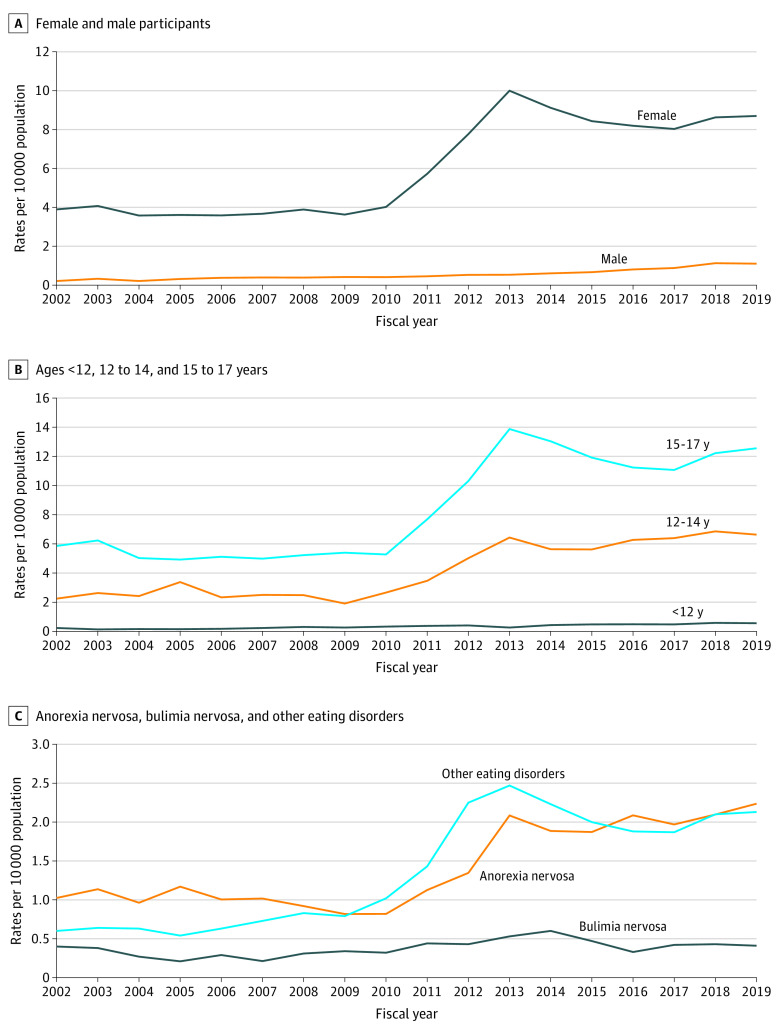
Annual Rates of Pediatric Eating Disorder Hospitalizations per 10 000 Population From April 1, 2002, to March 31, 2020, in Ontario, Canada

### Trends in Hospitalizations by Sex

Annual rates of eating disorder hospitalizations increased for male and female patients over the study period. Among male patients, the annual rate of hospitalizations increased 416%, from 0.2 hospitalizations per 10 000 population in 2002 to 1.1 hospitalizations per 10 000 population in 2019. For female patients, the annual rate of hospitalizations increased 123%, from 3.9 hospitalizations per 10 000 population in 2002 to 8.7 hospitalizations per 10 000 population in 2019 (Figure), with the largest absolute and relative increases observed from 2010 to 2013 (Table 2, Table 3, Table 4, and the Figure). The relative proportion of hospitalizations of male patients increased over the study period, from 5.4% of patients in 2002 to 11.8% of patients in 2019 (standardized difference, 0.23) (Table 2).

### Trends in Hospitalizations by Age Group

The annual hospitalization rates increased for all age groups over time, with the largest relative change among patients aged 12 to 14 years: a 196% increase was observed, from 2.2 hospitalizations per 10 000 population in 2002 to 6.6 hospitalizations per 10 000 population in 2019) (Figure, Table 3). Rates increased 140% for patients younger than 12 years (0.2 hospitalizations per 10 000 population in 2002 to 0.6 hospitalizations per 10 000 population in 2019) and 114% for patients aged 15 to 17 years (5.9 hospitalizations per 10 000 population in 2002 to 12.6 hospitalizations per 10 000 population in 2019). (Figure, Table 3, and Table 4). The proportion of hospitalizations of individuals aged 12 to 14 years increased significantly (standardized difference, 0.13) from 25.9% in 2002 to 31.9% in 2019, while the proportion of individuals aged 15 to 17 years old decreased (standardized difference, 0.12) from 67.9% in 2002 to 62.0% in 2019. No differences were observed among patients younger than 12 years (Table 2, Table 3, and Table 4).

### Trends in Hospitalizations by Eating Disorder Diagnostic Group

The largest relative increase in hospitalization rates occurred for eating disorder diagnoses other than anorexia or bulimia nervosa, including eating disorder not otherwise specified, unspecified eating disorders, pica, rumination, and feeding disorders of early childhood. Rates for this diagnostic group increased 255%, from 0.6 hospitalizations per 10 000 population in 2002 to 2.1 hospitalizations per 10 000 population in 2019, while rates for anorexia nervosa increased 118% from (1.0 hospitalizations per 10 000 in 2002 to 2.2 hospitalizations per 10 000 population in 2019), again, with the largest observed increases from 2010 to 2013. Hospitalization rates for bulimia remained relatively stable throughout the study period (Figure and Table 2). The proportion of hospitalizations with other eating disorder diagnoses increase from 29.5% in 2002 to 46.7% in 2019 (standardized difference, 0.36) while the proportion of hospitalizations with bulimia nervosa diagnoses decreased from 19.6% in 2002 to 8.6% in 2019 (standardized difference, 0.32). Unspecified eating disorders (3508 hospitalizations [30.6%]) and other eating disorders (839 hospitalizations [7.2%]) were the largest specific diagnostic codes in the other eating disorder diagnostic group (eFigure 2 in Supplement 1).

## Discussion

This cross-sectional study using large health administrative data sets found a 139% increase in pediatric eating disorder hospitalizations over a 17-year period in Ontario, Canada. The most substantial relative increases were observed among male patients (416%), younger adolescents (aged 12-14 years; 196%), and patients with eating disorders other than anorexia or bulimia nervosa (255%). Across all age groups, we observed significant absolute increases in eating disorder hospitalizations, with the largest increases for female patients and patients in midadolescence. These large increases and the observed changes in the proportion of hospitalizations of male patients, patients with bulimia nervosa, and patients with other eating disorders have implications for health system capacity and service provision.

Previous international research has reported increases in eating disorder service utilization among pediatric patients traditionally considered atypical. In the United States, the number of children younger than 12 years hospitalized with eating disorders increased from 1999 to 2006.^12^ Similarly, the number of individuals aged 10 to 14 years hospitalized with eating disorders increased in the United Kingdom from 2002 to 2011,^11^ and in Norway from 2010 to 2016.^24^ Studies have also shown increases in male patients seeking eating disorder care in Denmark from 1970 to 2008 and in the United Kingdom from 2002 to 2009.^10,33^ Moreover, diagnoses of eating disorders not otherwise specified increased in the United Kingdom from 2002 to 2009,^11^ while atypical anorexia nervosa, a subtype of eating disorders not otherwise specified, increased in Norway from 2010 to 2016.^24^ This cross-sectional study found that similar changes have been occurring in Canada over a more extended period.

Several factors may account for these findings, including an increase in the overall prevalence of eating disorders in the population, improved detection of illnesses by health care practitioners, or an increasing number of affected individuals seeking medical care due to reducing mental health stigma. Evaluating these possibilities effectively is challenging without provincial or national data on the prevalence of eating disorders outside of acute care settings over our study period.

An important consideration is the introduction of the *DSM-5* into clinical practice in 2013, which coincided with the highest annual pediatric eating disorder hospitalization rate in our study (5.1 hospitalizations per 10 000 population aged 5-17 years). The *DSM-5* underwent multiple revisions to address concerns that *DSM-IV* diagnostic criteria were not clinically applicable to many patients.^27,28^ These revisions included removing the amenorrhea and percentage body weight criteria from anorexia nervosa, broadening diagnostic criteria for anorexia nervosa to include behavioral as well as cognitive symptoms, reducing the required frequencies of binging or purging for bulimia nervosa and binge eating disorder, and expanding the diagnosis feeding and eating disorders of childhood into avoidant or restrictive food intake disorder, which includes individuals of all ages with restricted eating attributable to concerns other than body image. The use of these more inclusive revised criteria could have resulted in higher rates of eating disorder diagnoses in clinical settings in our study, especially among male patients, younger adolescents, and patients with other eating disorder diagnoses. Specifically, these new criteria may have led to more accurate diagnoses of anorexia nervosa, bulimia nervosa, and other specified eating disorders. However, they may have also contributed to diagnostic confusion, since unspecified eating disorder diagnoses peaked in 2013.

Previous research has indicated an increasing trend in mental health service utilization among children and youth (ages 10-24 years) in Ontario for diagnoses other than eating disorders over time.^34,35,36^ Pediatric patients seeking care for co-occurring mental health diagnoses could have had their eating disorders detected, potentially contributing to the observed increase in the number of co-occurring psychiatric diagnoses over time. Additionally, the province of Ontario launched a comprehensive mental health and addictions strategy during our study period, encompassing diverse initiatives, such as enhanced mental health literacy and increased funding to mental health agencies.^37^ These efforts likely contributed to increasing utilization of mental health services for both eating disorders and co-occurring mental health conditions.

### Implications

Understanding the changing characteristics of pediatric patients with eating disorders who are hospitalized is important for both clinical research and practice. Current evidence to inform the treatment of the patient subgroups historically considered atypical remains very limited. Future research should assess the applicability of specific eating disorder treatments to their care as well as patient and families’ experiences of care to ascertain whether existing services adequately meet their needs.

Our findings should be communicated to health care practitioners, as misperceptions about who is at risk for developing eating disorders contribute to delays in help seeking, misdiagnosis and appropriate referrals, particularly for male patients and younger patients.^38,39^ Such delays in appropriate eating disorder treatment may exacerbate serious medical concerns, including impaired growth, developmental delays, or reductions in bone density acquisition, that can be irreversible.^4^ Many health care practitioners also lack awareness of the dangers of eating disorders other than anorexia or bulimia nervosa.^40^ There is a pressing need for education to enhance health care practitioners’ knowledge on the prevalence, risks, and spectrum of pediatric eating disorder diagnoses across all ages and sexes. This education should include information on eating disorder psychiatric sequelae and co-occurring conditions, treatment goals, and evidence-based treatments.

### Limitations

This study has several limitations. First, the inclusion criteria were limited to hospitalizations with specific *ICD-10* and *DSM-IV* or *DSM-5* eating disorder diagnostic codes. This may have not captured patients with eating disorders with medical or psychiatric complications who were not assigned an eating disorder diagnostic code, potentially resulting in an underestimation of rates of pediatric eating disorder hospitalizations. The feasibility of applying the eating disorder codes used in this study to administrative data has been established, but they have not been formally validated.^29^ The sex variable in administrative data in Ontario reflects sex assigned at birth and does not account for patient-identified gender. Finally, because our study only included Ontario residents, results may not be generalizable to other provinces in Canada where pediatric eating disorder treatment is organized and funded differently.

## Conclusions

In this cross-sectional study of pediatric eating disorder hospitalizations, the utilization of inpatient pediatric eating disorder care in Ontario increased significantly from 2002 to 2019. This was accompanied by notable shifts in the demographic and clinical characteristics of hospitalized patients toward higher proportions of male patients, younger adolescents, and individuals with eating disorders aside from anorexia nervosa or bulimia nervosa. Pediatric patients who require hospitalization represent the most unwell or unstable children and adolescents with eating disorders at high risk for chronic morbidity and mortality. These patients require resource-intensive care to address their complex needs. The effectiveness of existing pediatric programs and treatments, particularly for patients with characteristics that have traditionally been considered atypical, is largely unknown and warrants further study. Health care practitioner education about the increasing diversity and psychiatric complexity of pediatric patients with eating disorders is needed to improve identification of these serious illnesses and support early access to appropriate care.

## Notes

Supplement 1. **eTable 1.** Diagnostic Codes Used to Capture Pediatric Eating Disorder Hospitalizations **eTable 2.** Classification of Co-Occurring Psychiatric Diagnostic Codes **eFigure 1.** Flowchart of Inclusion and Exclusion Criteria **eFigure 2.** Annual Frequencies of “Other” Eating Disorder Hospitalizations From 2002 to 2019

Supplement 2.
Data Sharing Statement
