# Supplementary material for: Pediatric Patients Hospitalized With Eating Disorders in Ontario, Canada, Over Time
Source: JAMA Netw Open. 2023 Dec 4;6(12):e2346012. doi: 10.1001/jamanetworkopen.2023.46012 (PMC10696484; doi:10.1001/jamanetworkopen.2023.46012)
Supplement: Supplement 1. — eTable 1. Diagnostic Codes Used to Capture Pediatric Eating Disorder Hospitalizations eTable 2. Classification of Co-Occurring Psychiatric Diagnostic Codes eFigure 1. Flowchart of Inclusion and Exclusion Criteria eFigure 2. Annual Frequencies of “Other” Eating Disorder Hospitalizations From 2002 to 2019 [file jamanetwopen-e2346012-s001.pdf]

Supplemental Online Content

Smith S, Charach A, To T, Toulany A, Fung K, Saunders N. Pediatric patients hospitalized with eating disorders in Ontario, Canada, over time. *JAMA Netw Open*. 2023;6(12):e2346012. doi:10.1001/jamanetworkopen.2023.46012

eTable 1. Diagnostic Codes Used to Capture Pediatric Eating Disorder Hospitalizations

eTable 2. Classification of Co-Occurring Psychiatric Diagnostic Codes

eFigure 1. Flowchart of Inclusion and Exclusion Criteria

eFigure 2. Annual Frequencies of “Other” Eating Disorder Hospitalizations From 2002 to 2019

This supplemental material has been provided by the authors to give readers additional information about their work.

© 2023 Smith S et al. *JAMA Network Open.*

eTable 1. Diagnostic codes used to capture pediatric eating disorder hospitalizations

**Eating Disorder Diagnostic Codes**	
**DAD[a] (ICD-10-CA[b]) April 1st, 2002 – March 30th, 2020**	
F50.0	Anorexia nervosa
F50.1	Atypical anorexia nervosa
F50.2	Bulimia nervosa
F50.3	Atypical bulimia nervosa
F50.4	Overeating associated with other psychological disturbances
F50.5	Vomiting associated with other psychological disturbances
F50.8	Other eating disorders
F50.9	Eating disorder specified
F98.2	Feeding disorder of childhood
F98.3	Pica
**OMHRS[c] (ICD-9-CM[d]) October 1st, 2005 – March 31st, 2019**	
307.1	Anorexia nervosa
307.50	Eating disorder not otherwise specified
307.51	Bulimia nervosa
307.52	Pica
307.53	Rumination disorder
307.54	Psychogenic vomiting
307.59	Other disorders of eating
Provisional diagnosis 10	DSM-5 Feeding and Eating Disorder
Provisional diagnosis 12	DSM-IV Eating Disorder
**OMHRS (ICD-10-CM[e]) April 1st, 2019 – March 31st, 2020**	
F50.0	Anorexia nervosa
F50.2	Bulimia nervosa
F50.8	Other eating disorders
F50.9	Eating disorder, unspecified
F98.21	Rumination disorder of infancy
F98.3	Pica of infancy and childhood

[a]DAD, Discharge Abstract Database; [b]ICD-10-CA, International Classification of Diseases, 10th edition with Canadian enhancements; [c]OMHRS, Ontario Mental Health Reporting System; [d]ICD-9-CM, International Classification of Diseases, Ninth Revision (clinical modification); [e]ICD-10-CM, International Classification of Diseases, Tenth Revision (clinical modification).

© 2023 Smith S et al. *JAMA Network Open.*

eTable 2. Classification of co-occurring psychiatric diagnostic codes

**Co-occurring Psychiatric Diagnostic Codes**	
**ICD-9-CM[a] (OMHRS[b])**	**ICD-10-CA[c] (DAD[d]) and ICD-10-CM[e] (OMHRS)**
DSM-IV 29190-31590, 99583	F06-F99

[a]ICD-9-CM, International Classification of Diseases, Ninth Revision (clinical modification); [b]OMHRS, Ontario Mental Health Reporting System; [c]ICD-10-CA, International Classification of Diseases, 10th edition with Canadian enhancements; [d]DAD, Discharge Abstract Database; [e]ICD-10-CM, International Classification of Diseases, Tenth Revision (clinical modification).

© 2023 Smith S et al. *JAMA Network Open.*

eFigure 1. Flowchart of inclusion and exclusion criteria.

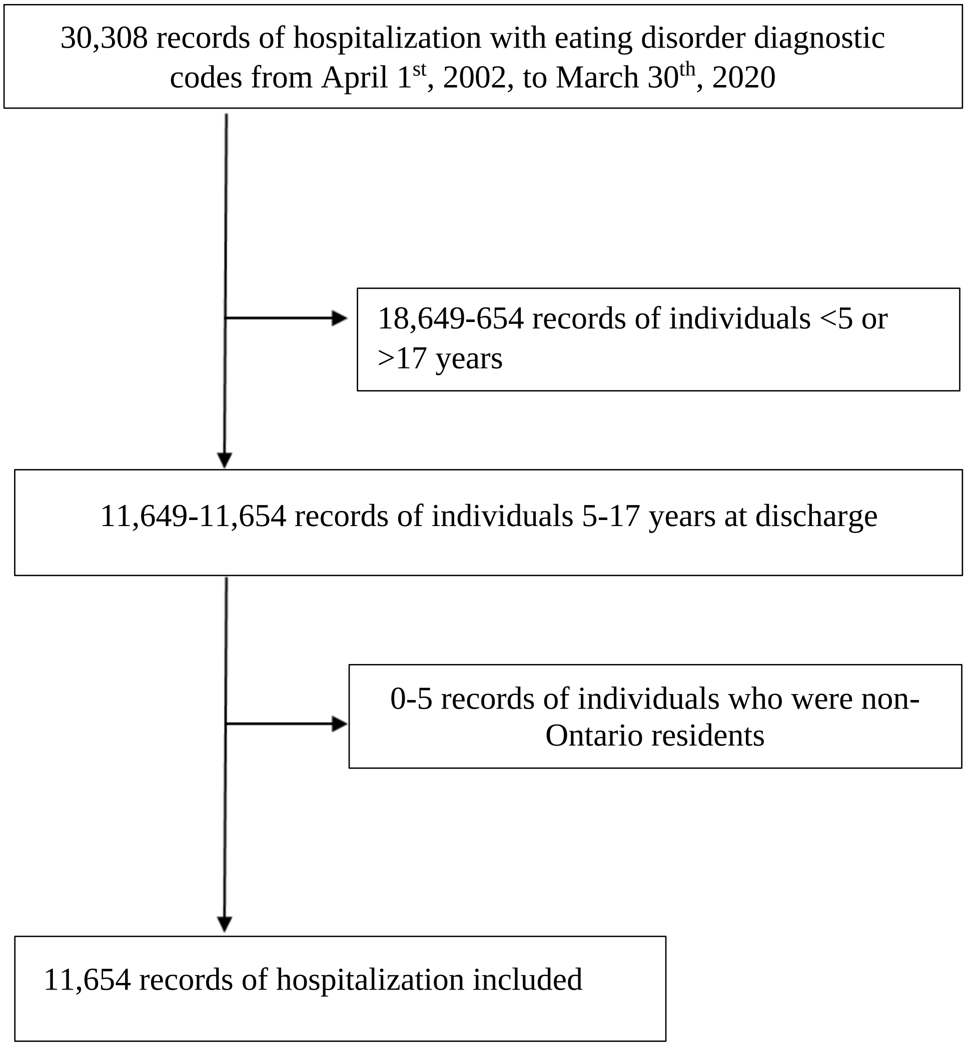

© 2023 Smith S et al. *JAMA Network Open.*

eFigure 2. Annual frequencies of "other" eating disorder hospitalizations from 2002 to 2019.

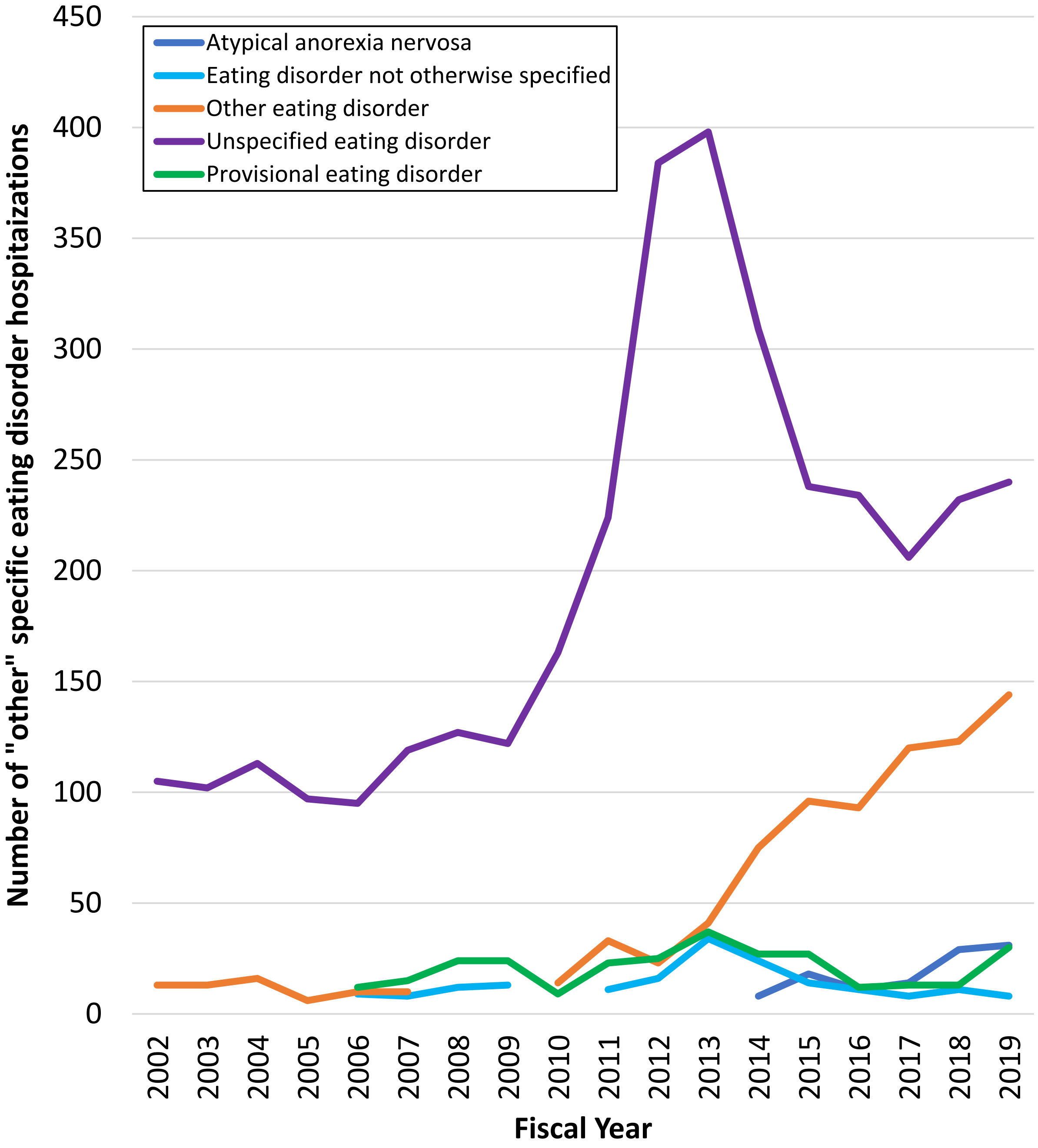

Cell sizes <6 suppressed due to institutional policy. Hospitalizations for atypical bulimia nervosa (n = 20), psychological overeating (n = 6), psychological vomiting (n = 78), feeding disorder of childhood (n = 84), pica (n = 77), rumination (n = 0) not included due to multiple annual small cell sizes. Diagnostic codes within "other" eating disorder categories are not mutually exclusive.

© 2023 Smith S et al. *JAMA Network Open.*
